# Synthesis of hexahydrofuro[3,2-c]quinoline, a martinelline type analogue and investigation of its biological activity

**DOI:** 10.1186/s40064-016-1890-5

**Published:** 2016-03-03

**Authors:** P.-Y. Chung, J. C.-O. Tang, C.-H. Cheng, Z.-X. Bian, W.-Y. Wong, K.-H. Lam, C.-H. Chui

**Affiliations:** State Key Laboratory of Chirosciences, Department of Applied Biology and Chemical Technology, The Hong Kong Polytechnic University, Hong Kong, China; Clinical Division, School of Chinese Medicine, Hong Kong Baptist University, Hong Kong, China; Department of Chemistry, Hong Kong Baptist University, Hong Kong, China

**Keywords:** Biological activity, *Candida albicans*, Hexahydrofuro[3,2-c]quinoline, Martinelline type analogue

## Abstract

**Background:**

Candida susceptibility commonly occurs in breast cancer patients. Of which, *Candida albicans* is considered as a common pathogen causing candidiasis. *Martinella iquitosensis* (Bignoniaceae) is one of the species belonged to *Martinella*, distributed widely in Amazon basin. Its root extract yielded two complex substituted tetrahydroquinolines, Martinelline and Martinellic acid which were the first natural non-peptide bradykinin receptor antagonists identified.

**Findings:**

In this study, a novel martinelline type analogue, named *2,3,3a,4,5,9b*-*hexahydro*-*8*-*phenoxy*-*4*-*(pyridin*-*2*-*yl)furo[3,2*-*c]quinoline*, was synthesized and its preliminary anticancer activity and antifungal potential were investigated. This compound showed potential anticancer activity against MDAMB-231 breast cancer cells. Meanwhile it could enhance the fungistatic activity of miconazole against *Candida albicans*.

**Conclusions:**

These findings provide an implication for the continue investigation and development of martinelline type analogues as therapeutic agents in the future.

## Background

Breast cancer patients are commonly susceptible to candidiasis. *Candida albicans* (*C. albicans*) is one of the opportunistic fungi especially observed in immunocompromised patients (Calderone and Fonzi 2001), such as cancer patients. Previous studies have investigated the prevalence of candidiasis in cancer patients. Among 845 women with multiple lymph node positive or metastatic breast carcinoma receiving high-dose chemotherapy and autologous bone marrow transplantation at Duke University Medical Center during 1992–1997, 29 of them (3.4 %) developed candidemia. Of which, 23 % of them were found to be infected with *C. albicans*. The mortality was highest for the women who were infected by *C. albicans* (71 %) (Gottfredsson et al. 2003). In 2005, 400 adult patients with the hematological malignancy, head neck or solid tumor (including breast cancer) were recruited randomly into the study on admission to the regional cancer center of the Norfolk and Norwich University Hospital. There were 56.8 % (227 of 400) of all cancer patients and 18.9 % (43 of 227) of those who had clinical and microbiological evidence of oral candidiasis. Among 269 yeast isolates recovered from 227 patients, *C. albicans* was the most common yeast (74 %) causing colonization and infection (Schelenz et al. 2011).

**Fig. 1:**
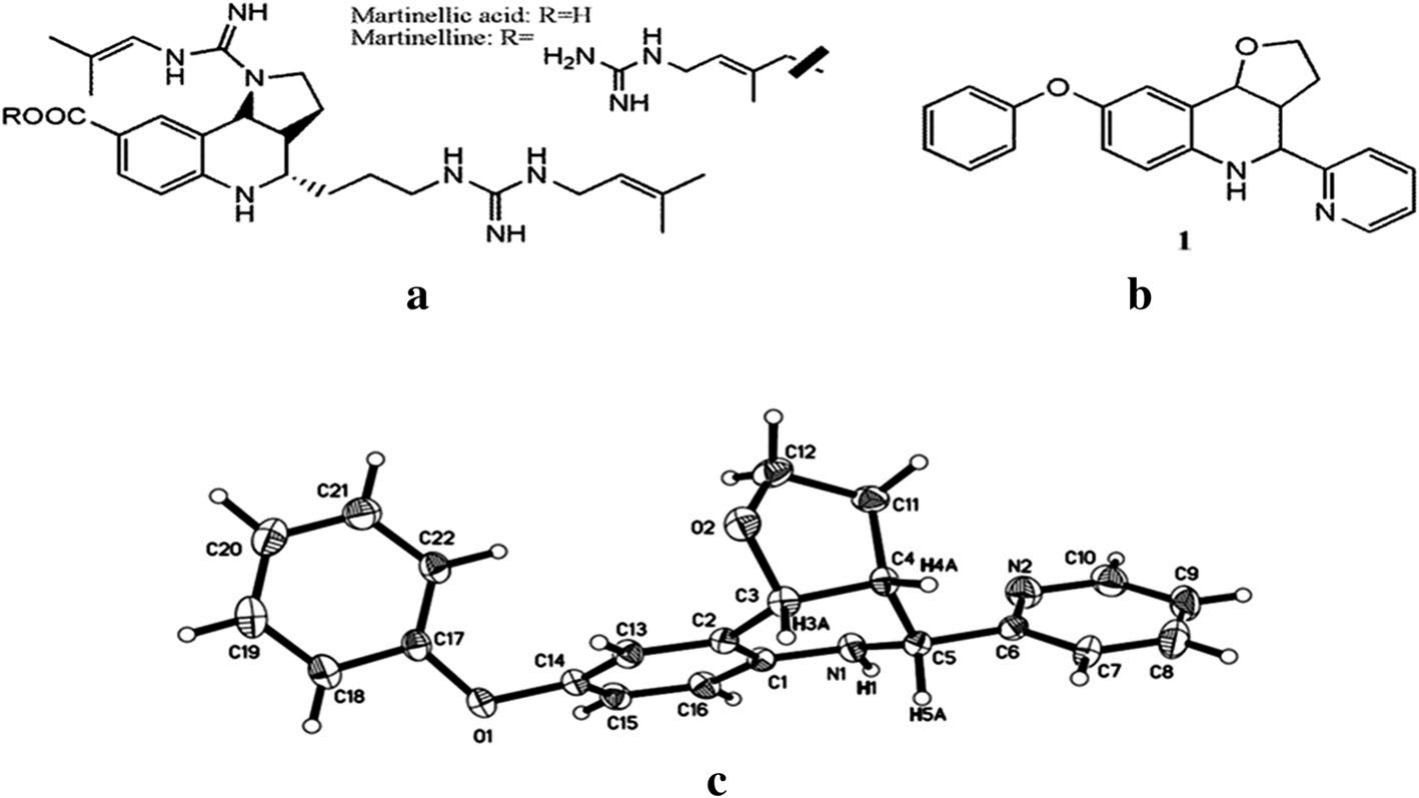
Structure of **a** Martinellic acid and Martinelline; **b** compound **1**; **c** MOLECULAR structure of compound **1**

*Martinella* can be used as indigenous medicine for eye disease caused by bacteria in various ethnolinguistic groups of some South American countries (Witherup et al. 1995). *Martinella iquitosensis* (Bignoniaceae), one of the species belonged to *Martinella*, is a tropical plant with dark purple corolla in South American and distributed widely in Amazon basin (Zuntini and Lohmann 2014). Its root extract yielded two complex substituted tetrahydroquinolines, Martinelline and Martinellic acid (Fig. 1a) which were the first natural non-peptide bradykinin receptor antagonists identified by Witherup et al. (1995). Afterwards, the studies on the synthesis of new compounds with this interesting core have been increased. For instance, hexahydro-2H-pyrano[3,2-c]quinolines were found to be a potent agent against pathogenic gram-negative bacteria and Magesh et al. discovered that one of the synthesized compounds exhibits good bacteriolytic activity against *Virbio vulnificus* and *Vibrio parahaemolticus* (Magesh et al. 2004). Another research group (Kantevari et al. 2011) also synthesized a series of hexahydro-2H-pyrano[3,2-c]quinolines and studied their activity against the *Mycobacrtium tuberculosisH37Rv*. Three of the synthesized compounds showed a comparable activity as ethambutol. In addition, it was reported that hexahydro-2H-pyrano[3,2-c]quinolines could be used as selective *σ*_1_ receptor ligand for the treatment of pain (Diaz et al. 2013).

Heterocyclic compounds are important candidates in the development of new class of structural entities for medicinal applications. Quinoline is a heterocyclic aromatic nitrogen containing compound characterized by a double-ring structure that has a benzene ring fused to pyridine at two adjacent carbon atoms (Keri and Patil 2014). Among these structures, tetrahydroquinoline derivatives demonstrated extensive biological activities. They included anticancer (Subramanian et al. 2011), antioxidant (Dorey et al. 2000) and antifungal (Vargas Méndez et al. 2010) activity. The construction and stereochemistry of the tricyclic ring system have also been recently reported (Calleja et al. 2014). In the previous years, we have prepared and studied some simple 2-subsituted terahydroquinoline alkaloid analogues as antitumor agents possessing notable cytotoxicity towards human Hep3B heptocellular carcinoma cells (Lam et al. 2013). In addition, we have reported the antimicrobial activity of some novel synthesized quinoline analogues (Lam et al. 2014; Chung et al. 2015). Inspired by unique core moiety of Martinelline and the reported promising bioactivity of tetrahydroquinolines and their related natural product analogues, we have designed compound **1** (Fig. 1b) and its potential biological activity was tested on MDAMB-231 breast cancer cells and *C. albicans*.

Strategically, we aimed to develop a simple and mild reaction pathway for the synthesis of compound **1** using one-pot multicomponent reaction with substituted aniline, aldehyde and alkene. Various catalysts, such as NbCl_5_ (da Silva et al. 2014), Fe_2_(SO_4_)·*x*H_2_O (Khan et al. 2011; Das et al. 2014), In(OTf)_3_ (Priestley et al. 2013) and BiCl_3_ (Kouznetsov et al. 2011) were reported to be effective for this reaction. Particularly, bismuth and its compounds are recognized as safe and green Lewis acid catalysts (Leonard et al. 2002; Mohan 2010). With increasing concern on the advancement of “green reaction” in last decade, the application of bismuth(III) compounds and their important roles in organic synthesis have been clearly addressed in recent researches (Gaspard-Iloughmane and Le Roux 2004; Bothwell et al. 2011; Ollevier 2013). Herein, we report a bismuth(III)-catalyzed synthesis of substituted tetrahydroquionlines.

## Methods

### General procedure for the synthesis of 2,3,3a,4,5,9b-hexahydro-8-phenoxy-4-(pyridin-2-yl)furo[3,2-c]quinoline (compound **1**)

All reagents were purchased from Sigma-Aldrich. All the synthesized compounds were characterized by ^1^H NMR, ^13^C NMR, mass spectrometry and X-ray crystallography. NMR spectra were recorded on a Bruker DPX400 Fourier transform spectrometer using CDCl_3_ as solvent unless otherwise specified. X-ray crystallographic analysis was performed by Bruker D8_VENTURE PHOTON 100. To a mixture of 2-Pyridinecarboxaldehyde (1.0 mol equiv) and 4-Phenoxyaniline (1.2 mol equiv) was added in solvent (2 mL) and stirred. Catalyst (0.2 mol equiv) and 2,3-Dihydrofuran (1.2 mol equiv) were then added. The reaction mixture was stirred at room temperature for 1.5 h. Solvent was then removed and the residue was extracted with dichloromethane. The organic layer was washed with 10 % Na_2_CO_3_ solution and water. Dried over anhydrous Na_2_SO_4_ and removed the solvent by rotary evaporator. The crude product was purified by silica gel column chromatography.

### Synthesis and characterization of compound **1**

^1^H NMR (400 MHz, CDCl_3_): *δ* 1.55–1.57 (m, 1H), 2.03–2.09 (m, 1H), 3.07–3.14 (m, 1H), 3.76–3.80 (m, 2H), 4.53 (s, 1H), 4.83 (d, 1H, *J* = 2.8 Hz), 5.30 (d, 1H, *J* = 8.0 Hz), 6.70 (d, 1H, *J* = 8.8 Hz), 6.84–6.98 (m, 1H), 7.00–7.06 (m, 3H), 7.11 (d, 1H, *J* = 2.4 Hz), 7.25–7.27 (m, 1H), 7.31–7.33 (m, 2H), 7.50 (d, 1H, *J* = 8.0 Hz), 7.75–7.77 (m, 1H), 8.64 (d, 1H, *J* = 4.4 Hz); ^13^C NMR (100 MHz, CDCl_3_): *δ* 24.40, 43.33, 57.67, 66.62, 75.91, 116.17, 117.60, 120.43, 120.56, 120.98, 122.18, 122.42, 123.85, 129.53, 136.79, 140.93, 149.10, 149.16, 158.66, 160.13; HRMS (ESI): Calcd. for C_22_H_21_N_2_O_2_ [M + H]^+^, 345.1598. found 345.1597. Melting point = 132.4–134.2 °C. Optimized yield = 26.8 %.

### [3-(4,5-dimethylthiazol-2-yl)-5-(3-carboxymethoxyphenyl)-2-(4-sulfophenyl)-2H-tetrazolium] (MTS) assay and cellular morphology

MDAMB-231 cells were obtained from American Type of Culture Collection. Changes in the cellular viability of compound **1**-treated cells were monitored using the MTS activity assay (20). Briefly, MDAMB-231 breast carcinoma cells were seeded at day 0. After 24 h, complete medium was changed and compound **1** was added at different concentrations (starting with 50 µM containing 0.1 % dimethyl sulfoxide (DMSO) as vehicle). Doxorubicin at 8 µM was used as a positive control. After 48 h of incubation, incubation medium was removed and fresh medium with MTS (Promega)/phenazine methosulfate as electron coupling agent mixed solution was added. Lastly, optical absorbance was determined at 490 nm using a microplate reader (Perkin Elmer Victor V) according to the user manual. Additionally, any morphological changes associated with compound **1** and doxorubicin treated breast cancer cells were recorded under an inverted microscope after fixing the cells with trichloroacetic acid and cellular protein was stained with sulforhodamine B after 24 h (Kok et al. 2007; Lam et al. 2015a, b).

### Determination of minimum inhibitory concentration (MIC) and sensitization assay

*Candida albicans* was obtained from American Type of Culture Collection. The MIC values of synthesized compound **1** and miconazole nitrate were determined by the broth dilution method. Various concentrations of compound **1** and miconazole were loaded from a starting concentration of 50 µM containing 0.1 % dimethyl sulfoxide (DMSO) as vehicle and they were diluted serially. DMSO (0.1 %) was used as a vehicle control. The fungal samples were then incubated at 37 °C for 48 h. The minimum concentrations of compound **1** and miconazole that induced a complete growth inhibition would be determined as their MIC values. For sensitization study, with compound **1** at 50 µM, miconazole was added at a starting concentration of 1.56, 0.78 and 0.39 µM respectively. After incubation, the fungal samples were treated with MTS/PMS as above (Lam et al. 2015a).

## Results and discussion

### Synthesis of compound **1**

We first screened different metal salts as catalyst for this reaction (Table 1). It was found that only metal(III) salts (Table 1, Entry 5–7) gave our desired compound **1** and, in particular, bismuth(III) nitrate pentahydrate provided the highest yield among all the selected catalysts. With such preliminary screening, we used Bi(NO_3_)_3_·5H_2_O as catalyst for further optimization of the reaction under various conditions aiming to enhance the product yield (Table 2). It is noted that addition of catalyst in 0.2 mol equivalent and the reaction carried in ethanol (Table 2, Entry **7**) provided the best yield. With the present work, further investigation is ongoing to develop a greener and more effective reaction for the synthesis of compound **1**.

**Table 1:** Catalyst screening to optimize the product yield

Entry	Catalyst	Yield (%)
1	MnCl_2_·4H_2_O	N.D.
2	FeSO_4_·7H_2_O	N.D.
3	CoCl_2_·6H_2_O	N.D.
4	CuSO_4_·5H_2_O	N.D.
5	Fe(NO_3_)_3_·9H_2_O	16.5
6	BiCl_3_	9.9
7	Bi(NO_3_)_3_·5H_2_O	19.6

All the reactions were carried out at room temperature in acetonitrile (ACN) for 1.5 h

*N.D.* not detected

**Table 2:** Optimization on the product yield using Bi(NO_3_)_3_·5H_2_O

Entry	Solvent	Reaction time (h)	Yield (%)
1^a^	ACN	1.5	6.8
2^b^	ACN	1.5	10.2
3	ACN	1.5	19.6
4	CH_2_Cl_2_	1.5	17.1
5	Water	1.5	9.1
6^c^	Water	1.5	12.0
7	Ethanol	1.5	22.2
8^d^	ACN	1.5	21.8
9	Ethanol	0.5	26.8
10	Ethanol	3	25.3
11	Ethanol	7	18.6

All the reactions were carried out at room temperature and catalyst (0.2 mol equiv) was added

^a^Catalyst (0.05 mol equiv) was added

^b^Catalyst (0.1 mol equiv) was added

^c^0.1 M nitric acid was used and no metal catalyst was added

^d^The reaction was carried out under reflux

### Anticancer activity of compound **1**

**Fig. 2:**
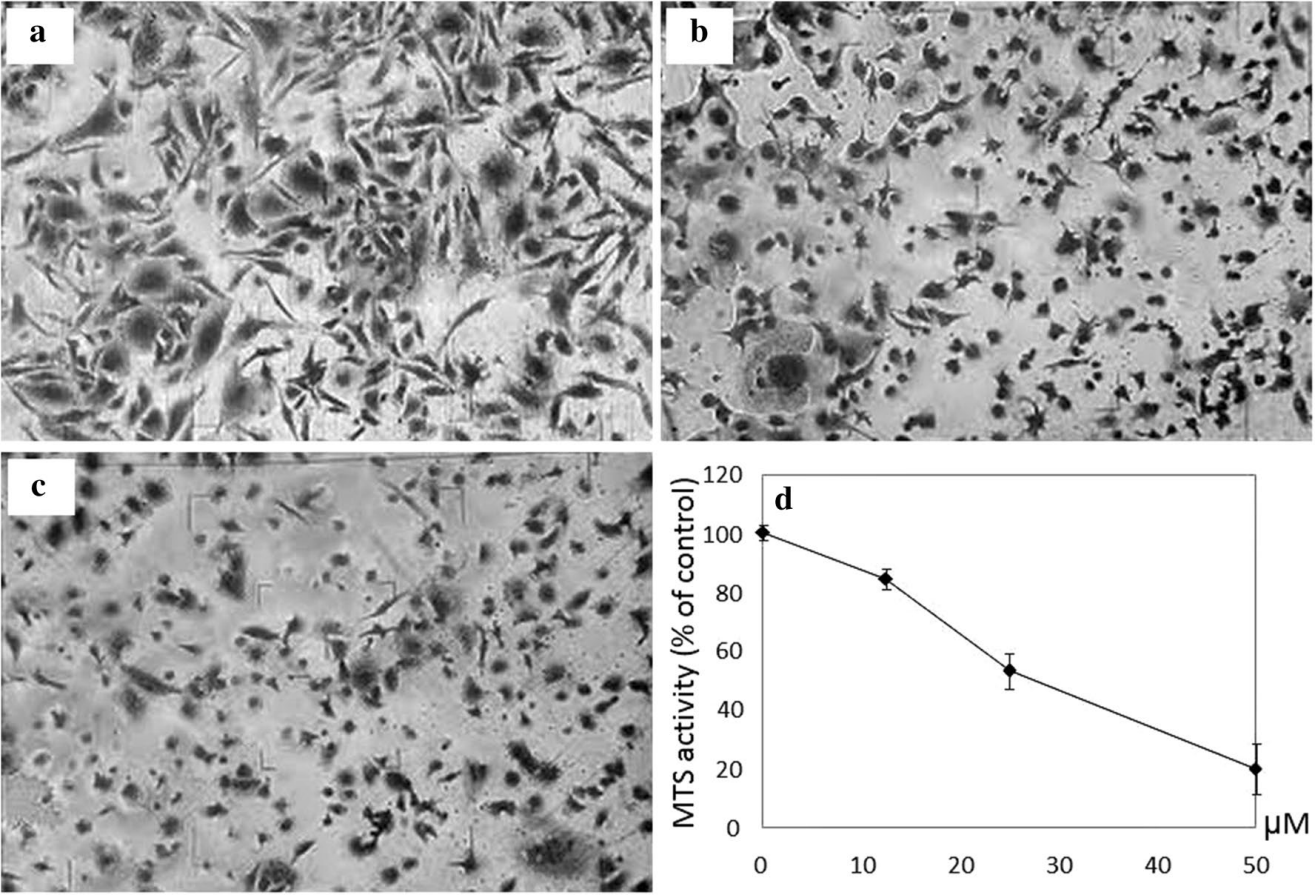
**a**–**c** Cellular morphology of MDAMB-231 cells after 24 h: **a** Untreated control; **b** compound **1** at 50 µM; **c** Doxorubicin at 8 µM as positive reference; and **d** MTS activity assay to determine the effect of compound **1** on MDAMB-231 cells after 48 h. Reported results represent the mean ± SD from triplicate tests. This figure shows a representative experiment taken from three independent experiments giving similar results

Compound **1** at 50 µM (~17 µg/ml) could readily induce cell death on MDAMB-231 cells with significant cellular morphological changes when compared with the untreated control (Fig. 2a) such as cell rounding and shrinkage (Fig. 2b) which were similar to those from the positive reference, doxorubicin (Fig. 2c) at 8 µM after 24 h. As shown in Fig. 2d, a dose dependent cytotoxicity of compound **1** on MDAMB-231 breast cancer cells was observed after a 48 h of incubation.

### MIC of compound **1**

**Fig. 3:**
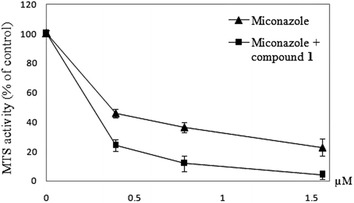
MTS activity assay to determine the effect of miconazole alone and micronazole with compound **1** (50 µM) on *C. albicans*. Reported results represent the mean ± SD from triplicate tests. This figure shows a representative experiment taken from three independent experiments giving similar results

The MIC value of miconazole on *C. albicans* was found to be 3 µM. Compound **1**, however, did not exhibit antifungal activity on *C. albicans* of up to 50 µM. However, we found that compound **1** could enhance the antifungal activity of miconazole on *C. albicans*. In the subsequent tests, compound **1** was added simultaneously with different concentrations of miconazole. As shown in Fig. 3, compound 1 could significantly potentiate the antifungal action of miconazole. Recently, we have shown that corilagin could sensitize Hep3B hepatoma cells to cisplatin and doxorubicin (Gambari et al. 2014). Here we suggest that compound **1** at 50 µM could significantly improve the fungistatic property of miconazole against *C. albicans*.

## Conclusions

In this work, the synthesis and preliminary in vitro biological application of a novel martinelline type analogue, named *2,3,3a,4,5,9b*-*hexahydro*-*8*-*phenoxy*-*4*-*(pyridin*-*2*-*yl)furo[3,2*-*c]quinoline*, were described. Interestingly, this compound showed potential anticancer activity against MDAMB-231 breast cancer cells and it could simultaneously potentiate the fungistatic activity of miconazole against a common human pathogenic fungus, *C. albicans*. As the obtained compound **1** consists of three chiral centres, there should be eight stereoisomers exist. Further work will be carried out to isolate each isomer and investigate their individual potential biological activity in order to elucidate if chirality is important in this group of compounds from the pharmaceutical point of view.

## Abbreviations

*C. albicans*: *Candida albicans*
DMSO: dimethyl sulfoxide
MIC: minimum inhibitory concentration
MTS: [3-(4,5-dimethylthiazol-2-yl)-5-(3-carboxymethoxyphenyl)-2-(4-sulfophenyl)-2H-tetrazolium]
